# Booster Dose Vaccination and Dynamics of COVID-19 Pandemic in the Fifth Wave: An Efficient and Simple Mathematical Model for Disease Progression

**DOI:** 10.3390/vaccines11030589

**Published:** 2023-03-03

**Authors:** Thitiya Theparod, Pannathon Kreabkhontho, Watchara Teparos

**Affiliations:** 1 Department of Mathematics, Mahasarakham University, Maha Sarakham 44150, Thailand; 2 Department of General Science, Faculty of Science and Engineering, Chalermphrakiat Sakon Nakhon Province Campus, Kasetsart University, Sakon Nakhon 47000, Thailand

**Keywords:** COVID-19, mathematical modeling, Omicron, simulation, vaccination

## Abstract

Background: Mathematical studies exploring the impact of booster vaccine doses on the recent COVID-19 waves are scarce, leading to ambiguity regarding the significance of booster doses. Methods: A mathematical model with seven compartments was used to determine the basic and effective reproduction numbers and the proportion of infected people during the fifth wave of COVID-19. Using the next-generation matrix, we computed the effective reproduction parameter, Rt. Results: During the fifth COVID-19 wave, the basic reproductive number in Thailand was calculated to be $R_{0}$= 1.018691. Analytical analysis of the model revealed both local and global stability of the disease-free equilibrium and the presence of an endemic equilibrium. A dose-dependent decrease in the percentage of infected individuals was observed in the vaccinated population. The simulation results matched the real-world data of the infected patients, establishing the suitability of the model. Furthermore, our analysis suggested that people who had received vaccinations had a better recovery rate and that the death rate was the lowest among those who received the booster dose. The booster dose reduced the effective reproduction number over time, suggesting a vaccine efficacy rate of 0.92. Conclusion: Our study employed a rigorous analytical approach to accurately describe the dynamics of the COVID-19 fifth wave in Thailand. Our findings demonstrated that administering a booster dose can significantly increase the vaccine efficacy rate, resulting in a lower effective reproduction number and a reduction in the number of infected individuals. These results have important implications for public health policymaking, as they provide useful information for the more effective forecasting of the pandemic and improving the efficiency of public health interventions. Moreover, our study contributes to the ongoing discourse on the effectiveness of booster doses in mitigating the impact of the COVID-19 pandemic. Essentially, our study suggests that administering a booster dose can substantially reduce the spread of the virus, supporting the case for widespread booster dose campaigns.

## 1. Introduction

Coronavirus disease 2019 (COVID-19) is an infectious respiratory disease caused by the novel coronavirus, SARS-CoV-2, which was first identified in Wuhan, China, in December 2019 [1,2,3,4,5,6,7]. It has been declared a global pandemic by the World Health Organization (WHO) and has significantly impacted public health, economies, and daily life worldwide. The virus spreads through respiratory droplets when an infected person talks, coughs, or sneezes and can also be transmitted through close contact with an infected person or by touching surfaces contaminated with the virus. Due to the severity and rapid spread of the disease, public health officials and governments worldwide have implemented a range of measures to control its spread. These measures include social distancing, wearing masks, frequent hand washing, and vaccination campaigns. Several vaccines have been authorized for emergency use worldwide, and ongoing research is focused on improving vaccine efficacy, developing treatments, and understanding the long-term health effects of COVID-19 [8].

COVID-19 still remains a major problem in some countries, and the possibility of new immunity-evading variations poses a hazard to global health, especially for immunosuppressed individuals [1,2]. Nonetheless, in several countries, COVID-19 has reached an endemic stage; as of the beginning of 2022, Delta and Omicron are the two most prevalent variants [3]. From the initial day of the COVID-19 outbreak until 6 December 2022, more than 645 million confirmed infections and more than 6.6 million confirmed deaths have been recorded globally [3,4].

Vaccination is considered an essential tool for the long-term management of the COVID-19 outbreak [5,6]. Strategies for containing the virus include prohibiting travel from highly infected areas, social isolation of infected people, lockdown policies, self-quarantine of exposed people, mandatory use of face masks or face coverings, and strict adherence to all socially conscious and prevention strategies. As of 6 December 2022, 68.5% of the world’s population had been vaccinated against COVID-19 with at least one dose. Over 10 billion doses have been delivered worldwide [7]. However, in low-income countries, the vaccination rate remains very low, and some people continue to doubt the benefits and effectiveness of vaccines [8,9,10]. Because of these issues, independent studies are specifically designed to offer evidence of the usefulness of vaccination programs.

Mathematical modeling provides valuable insight into the spread of the disease and the effectiveness of potential responses. Indeed, understanding the epidemic and forecasting how long an epidemic will endure and how the disease will spread in general is made possible by infectious disease modeling [11]. Essential characteristics, including the dynamics of disease transmission, the number of people infected, the number of people hospitalized, and the number of people who have recovered or died, can be estimated using the compartmental modeling methodology [12].

Before the development of COVID-19 vaccines, mathematical models were used to analyze the effects of non-pharmaceutical measures, such as social isolation, mask usage, improved hygiene practices, quarantine, and lockdown. Since vaccines are now widely available as a control tool, epidemiologists include this novel intervention in their models to better explain the dynamic characteristics of the disease and to make clear policy recommendations to public health authorities. Immunization of networks with limited knowledge and temporary immunity can be effectively employed to model the spread of infectious diseases through complex networks, i.e., a system of interconnected individuals or entities that can transmit infectious diseases. The concept of immunizing a network involves identifying specific individuals within the network who are the most likely to transmit the disease and vaccinating them to reduce the overall spread of the disease. However, there may be limited knowledge about the network in many cases, including information about the individuals most likely to transmit the disease.

Additionally, the immunity generated by vaccination may be temporary, meaning that individuals may become susceptible to the disease again over time. It is therefore important that such models take into account the knowledge of the network and the temporary nature of immunity. By developing and testing these frameworks through mathematical modeling, a better understanding of the spread of infectious diseases and develop effective public health interventions to limit their impact. The most popular models used to characterize COVID-19 are community susceptible-infected-removal (SIR) models that involve a system of differential equations or stochastic discrete mathematics [13]. Mixed SI(R) epidemic dynamics in random graphs with general degree distributions could also be an effective tool to simulate the spread of the disease in random graphs, which have varying degrees of connectivity. Some agent-based models created to simulate COVID-19 involve stochastic modeling of virus transmission via structured networks between people [14]. A recent scoping review revealed that, although SEIR-based vaccines are mature and comprehensive, vaccination and the emergence of mutant strains require further investigation [15].

Similar to many other countries, Thailand is heavily dependent on tourism as a source of foreign currency and economic growth. Even after COVID-19, when it comes to vacation spots, Thailand has not lost any of its appeal [16,17]. This success may be partly attributed to Thailand’s highly developed healthcare delivery system and almost universal health insurance coverage [18]. As most countries and airlines have less stringent travel rules, it is reasonable to assume that many people from all over the world will seek to come here, vaccinated and unvaccinated. We infer that there is a dearth of rigorous scientific research on the spread of COVID-19 in Thailand and that, to our knowledge, no studies have used mathematical modeling to account for the latest wave of COVID-19 and the effect of the booster vaccine dose.

This study aims at developing a mathematical model that can effectively predict the COVID-19 progression dynamics in the setting of multiple vaccinations. To this end, theoretical and numerical analysis of the dynamics of the COVID-19 epidemic has been conducted to answer critical questions regarding the impact of different vaccination doses on several outcomes associated with COVID-19. Finally, we tested our model in a real-world data in Thailand and discussed the influence of vaccination on disease dynamics in the fifth wave, analyzing the presence of endemic equilibrium points and the stability of disease-free equilibrium.

## 2. Mathematical Model and Analysis

### 2.1. Model Descriptions

**Figure 1:**
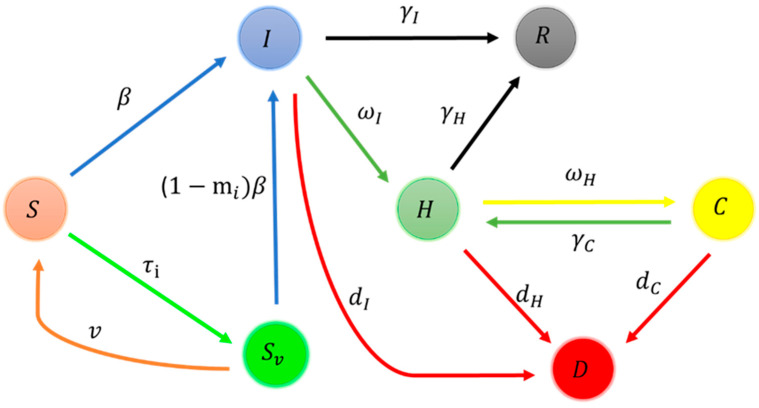
Schematic diagram of the model for disease transmission.

We propose a seven-compartment mathematical model to analyze the dynamics of the COVID-19 pandemic (Figure 1).

Here, the total population was divided into seven compartments, representing the fraction of the susceptible population (S), the fraction of the vaccinated population ($S_{v}$), the fraction of infectious individuals under home quarantine and self-care (I), the fraction of hospitalized individuals (H), the fraction of critically infected individuals who are treated in the ICU (C), the fraction of recovered population (R), and the fraction of dead population (D), respectively. Susceptible individuals acquired infection when they came into contact with an infectious individual at rate β, and parameter $m_{i}$ denotes the vaccine efficiency of the ith dose of vaccination (Table 1). We assumed that individuals in the H and C classes were isolated from the population and therefore would have a negligible role in transmitting the disease. Due to the vaccine efficacy $m_{i}$, individuals in $S_{v}$ compartment are relatively less infected than the fully susceptible ones. The majority of vaccinations authorized by the World Health Organization are administered in two doses, with an average suggested time gap between doses. Individuals in I and H will recover from the disease at the rate of $\gamma_{I}$ and $\gamma_{H},$ respectively.

The total population is assumed to be close; therefore, the total population at time *t* is denoted by *N*(*t*), where

$$N(t)=S(t)+S_{v}(t)+I(t)+H(t)+C(t)+R\left(t)+D(t\right)=1$$

A system of nonlinear ordinary differential equations yields the following mathematical model.

(1) $$\begin{array}{l} \frac{dS}{dt}=vS_{v}\left(t\right)-\beta S\left(t\right)I\left(t\right)-\tau_{i}S\left(t\right)\\ \frac{dSv}{dt}=\tau_{i}S\left(t\right)-\left(1-m_{i}\right)\beta S_{v}\left(t\right)I\left(t\right)-vS_{v}\left(t\right)\\ \frac{dI}{dt}=\beta S\left(t\right)I\left(t\right)+\left(1-m_{i}\right)\beta S_{v}\left(t\right)I\left(t\right)-\gamma_{I}I\left(t\right)-\omega_{I}I\left(t\right)-d_{I}I\left(t\right)\\ \frac{dH}{dt}=\omega_{I}I\left(t\right)+\gamma_{C}C\left(t\right)-\omega_{H}H\left(t\right)-\gamma_{H}H\left(t\right)-d_{H}H\left(t\right)\\ \frac{dC}{dt}=\omega_{H}H\left(t\right)-\gamma_{C}C\left(t\right)-d_{C}C\left(t\right)\\ \frac{dR}{dt}=\gamma_{I}I\left(t\right)+\gamma_{H}H\left(t\right)\\ \frac{dD}{dt}=d_{I}I(t)+d_{H}H(t)+d_{C}C(t) \end{array}$$

This model has initial conditions $N\left(0\right)\geq 0,S\left(0\right)\geq 0,S_{v}\left(0\right)\geq 0,I\left(0\right)\geq 0,H\left(0\right)\geq 0,C\left(0\right)\geq 0,R(0)\geq 0$ and D(0)≥0.

All model parameters are listed in Table 1.

### 2.2. Disease-Free Equilibrium

**Theorem** **1.** *A disease-free equilibrium state (DFE) of the model (1) exists at the point*$E_{0}=\left(S^{0},S_{v}^{0},I^{0},H^{0},C^{0},R^{0},D^{0}\right)=\left(S^{0}=\frac{vS_{v}^{0}}{\tau_{i}},S_{v}^{0}=S_{v}^{0},0,0,0,0,0\right)$*and the endemic equilibrium point exists at*$\left(S^{*},S_{v}^{*},I^{*},H^{*},C^{*},R^{*}\right)=(S^{*},S_{v}^{*},I^{*},H^{*}=\frac{\left(\omega_{I}+\gamma_{I}\right)I^{*}+\gamma_{c}C^{*}}{\left(\omega_{H}+d_{H}\right)},C^{*},R^{*})$*, where*$S^{*},S_{v}^{*},I^{*},C^{*}$, $R^{*}$ > 0 *are arbitrary*.

**Proof.**  The above expressions are deduced by equating all derivatives mentioned in the set of system Equation (1) to zero. The detailed proof can be found in Appendix A.1. □

### 2.3. Next-Generation Matrix and Basic Reproduction Number

The basic reproduction number is a crucial threshold for the analysis of infectious diseases. It determines whether a disease will die or persist in the population [19,20]. The basic reproduction number ($R_{0})$ is defined as the average number of secondary cases that one primary case of an infected individual would produce in an entirely susceptible population. If $R_{0}$ is more than 1, the DFE is unsustainable, which allows for the index infection to culminate in several infections and the emergence of an epidemic. Contrarily, if $R_{0}$ is smaller than 1, the DFE is locally asymptotically stable, and the infection cannot propagate throughout the population, the situation is sustained.

In this paper, we used the method of a next-generation matrix to determine $R_{0}$ [21]. We let $x=(I,H,C,{)}^{T},$ and $\frac{dx}{dt}=$ $F\left(x\right)-V\left(x\right),$ where F(X) represents the rate of new infection, and V(x) represents the rate of movements into and out of each compartment.

$$F(x)=\left[\begin{array}{l} \beta SI+(1-m_{i})\beta S_{v}I\\ 0\\ 0 \end{array}\right]$$

and

$$V(X)=\left[\begin{array}{l} (\gamma_{I}+\omega_{I}+d_{I})I\\ -\omega_{I}I-\gamma_{C}C+(\omega_{H}+\gamma_{H}+d_{H})H\\ -\omega_{H}H+(\gamma_{C}+d_{C})C \end{array}\right]$$

If f and v represent the Jacobian matrices of F and V, as determined by the DFE, respectively, we have

$$f=\left[\begin{array}{ccc} \beta +(1-m)\beta & 0 & 0\\ 0 & 0 & 0\\ 0 & 0 & 0 \end{array}\right]$$

and

$$v=\left[\begin{array}{ccc} \gamma_{I}+\omega_{I}+d_{I} & 0 & 0\\ -\omega_{I} & \omega_{H}+\gamma_{H}+d_{H} & -\gamma_{C}\\ 0 & -\omega_{H} & \gamma_{C}+d_{C} \end{array}\right]$$

Then

$$v^{-1}=\left[\begin{array}{ccc} \frac{1}{\gamma_{I}+\omega_{I}+d_{I}} & 0 & 0\\ \frac{\omega_{I}\left(\gamma_{C}+d_{C}\right)}{\left(\gamma_{I}+\omega_{I}+d_{I}\right)(\gamma_{C}+d_{C})(\omega_{H}+\gamma_{H}+d_{H})} & \frac{\gamma_{C}+d_{C}}{\left(\gamma_{C}+d_{C})(\omega_{H}+\gamma_{H}+d_{H}\right)} & \frac{\gamma_{C}}{\left(\gamma_{C}+d_{C})(\omega_{H}+\gamma_{H}+d_{H}\right)}\\ \frac{\omega_{I}\omega_{H}}{\left(\gamma_{I}+\omega_{I}+d_{I}\right)(\gamma_{C}+d_{C})(\omega_{H}+\gamma_{H}+d_{H})} & \frac{\omega_{H}}{\left(\gamma_{C}+d_{C})(\omega_{H}+\gamma_{H}+d_{H}\right)} & \frac{\omega_{H}+\gamma_{C}+d_{C}}{\left(\gamma_{C}+d_{C})(\omega_{H}+\gamma_{H}+d_{H}\right)} \end{array}\right]$$

Therefore, the next-generation matrix fv^−1^ is

$$fv^{-1}=\left[\begin{array}{ccc} \frac{\beta S^{*}+(1-m_{i})\beta S_{v}^{*}}{\gamma_{I}+\omega_{I}+d_{I}} & 0 & 0\\ 0 & 0 & 0\\ 0 & 0 & 0 \end{array}\right]$$

$R_{0}$ represents the spectral radius of the matrix $fv^{-1}$,

$$\rho (fv^{-1})=\frac{\beta }{\gamma_{I}+\omega_{I}+d_{I}}=R_{0}$$

### 2.4. Stability Analysis

Initially, we performed a stability analysis at the disease-free and endemic equilibrium points. The statement of the outcomes is as follows:

**Theorem** **2.** *The disease-free equilibrium point,*$E^{0},$*is stable if*$R_{0}<1$*, whereas*$E^{0}$*is unstable if*$R_{0}>1$.

**Proof.**  The Jacobian matrix of model (1) evaluated at $E^{0}$ is. □

$$JE^{0}=\left[\begin{array}{cccc} -\tau_{i} & v & -\beta & \begin{array}{cc} 0 & \;\;\;\;\;\;\;\;\;\;\;\;\;\;\;\;\;\;\;\;\;\;\;\;\;\;0 \end{array}\\ \tau_{i} & -v & 0 & \begin{array}{cc} 0 & \;\;\;\;\;\;\;\;\;\;\;\;\;\;\;\;\;\;\;\;\;\;\;\;\;\;\;0 \end{array}\\ 0 & 0 & \beta -(\gamma_{I}+\omega_{I}+d_{I}) & \begin{array}{cc} 0 & \;\;\;\;\;\;\;\;\;\;\;\;\;\;\;\;\;\;\;\;\;\;\;\;\;\;\;0 \end{array}\\ \begin{array}{l} 0\\ 0 \end{array} & \begin{array}{l} 0\\ 0 \end{array} & \begin{array}{l} \omega_{I}\\ 0 \end{array} & \begin{array}{cc} \begin{array}{l} -(\omega_{H}+\gamma_{H}+d_{H})\\ \;\;\;\;\;\;\;\;\;\;\;\;\omega_{H} \end{array} & \begin{array}{l} \gamma_{C}\\ -(\gamma_{C}+d_{C}) \end{array} \end{array} \end{array}\right]$$

and the characteristic polynomial is $det(J_{E^{0}}-\lambda I)=0.$ Solving this polynomial, the eigenvalues are given by

$$\det(J_{DFE}-\lambda I)=\left[\begin{array}{cccc} -\tau_{i}-\lambda_{1} & v & -\beta & \begin{array}{cc} 0 & \;\;\;\;\;\;\;\;\;\;\;\;\;\;\;\;\;\;\;\;\;\;\;\;\;\;0 \end{array}\\ \tau_{i} & -v-\lambda_{2} & 0 & \begin{array}{cc} 0 & \;\;\;\;\;\;\;\;\;\;\;\;\;\;\;\;\;\;\;\;\;\;\;\;\;\;\;0 \end{array}\\ 0 & 0 & \beta -(\gamma_{I}+\omega_{I}+d_{I})-\lambda_{3} & \begin{array}{cc} 0 & \;\;\;\;\;\;\;\;\;\;\;\;\;\;\;\;\;\;\;\;\;\;\;\;\;\;\;0 \end{array}\\ \begin{array}{l} 0\\ 0 \end{array} & \begin{array}{l} 0\\ 0 \end{array} & \begin{array}{l} \omega_{I}\\ 0 \end{array} & \begin{array}{cc} \begin{array}{l} -(\omega_{H}+\gamma_{H}+d_{H})-\lambda_{4}\\ \;\;\;\;\;\;\;\;\;\;\;\;\omega_{H} \end{array} & \begin{array}{l} \gamma_{C}\\ -(\gamma_{C}+d_{C})-\lambda_{5} \end{array} \end{array} \end{array}\right]$$

$$\det(J_{DFE}-\lambda I)=\left(-\tau_{i}-\lambda_{1}\right)\left(-v-\lambda_{2}\right)\left(\beta -(\gamma_{I}+\omega_{I}+d_{I})-\lambda_{3}\right)\left(-(\omega_{H}+\gamma_{H}+d_{H})-\lambda_{4}\right)\left(-(\gamma_{C}+d_{C})-\lambda_{5}\right)$$

We get

$$\lambda_{1}=-\tau_{i}<0$$

$$\lambda_{2}=-v<0$$

$$\lambda_{4}=-(\omega_{H}+\gamma_{H}+d_{H})<0$$

$$\lambda_{5}=-(\gamma_{C}+d_{C})<0$$

and

$$\lambda_{3}=\frac{\beta }{\left(\gamma_{I}+\omega_{I}+d_{I}\right)}-\frac{\left(\gamma_{I}+\omega_{I}+d_{I}\right)}{\left(\gamma_{I}+\omega_{I}+d_{I}\right)}=R_{0}-1<0$$

All eigenvalues are zero or negative. Hence, $E^{0}$ is stable.

**Theorem** **3.** *The endemic equilibrium point,* $E^{*},$ *of model 1 is stable if* $R_{0}>1$ *, in the case of no vaccination rate, i.e.,* $\tau_{i},v=0$ *, where* $0<S_{v}<1$ *and* $\frac{\beta I+\left(d_{I}+\gamma_{I}+\omega_{I}\right)}{\beta }<S<I-\left(1-m_{i}\right)\left(S_{v}-I\right).$

**Proof.**  The mathematical expression of eigenvalues of the Jacobian matrix of the model (1) is tedious. The detailed derivation can be found in Appendix A.2. □

## 3. Numerical Simulations and Fitting

### Parameter Estimation

**Figure 2:**
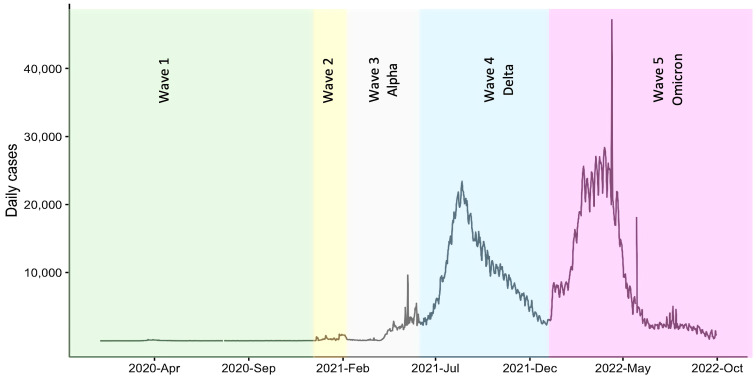
Course of COVID-19 in Thailand from the beginning of the pandemic.

Our analysis used information provided by the DDC [22]. Figure 2 shows the spread of COVID-19 in Thailand. The fourth and fifth waves, as can be seen, had a very high incidence rate. In the vaccination rates for the fourth wave, the first dose, second dose, and third dose were 77%, 70%, and 11%, respectively. In the vaccination rates for the fifth wave, the first dose, second dose, and third dose were 81%, 77%, and 42%, respectively.

Since the fifth wave had the highest immunization rate and we had information on the first, second, and third doses, we concentrated on modeling the fifth wave progression. The estimation of the model parameters is fundamental for obtaining accurate numerical results. In this study, the parameter estimation was based on actual data from COVID-19 patients confirmed in Thailand during the fifth wave from 3 January to 9 July 2022. Some of the parameters were calculated from the data as follows: The total population of Thailand is approximately N = 66,000,000. Furthermore, at the beginning of the fourth wave of the epidemic in Thailand, there were 2,927 symptomatic patients, 33,114 hospitalizations, and 704 admissions to the ICU. Therefore, assuming the initial number is 65,960,334, the initial population is assumed to be N(t)=1. Some of the remaining parameters were evaluated using the mathematical model (1). In our study, we also used scientifically reported information regarding COVID-19 transmission mechanisms. DDC [22,23] provided information about $\gamma_{I}$, $\gamma_{H}$, $\omega_{H}$, $\gamma_{C}$, $d_{C}{,d}_{H},$ and $\tau_{i}$. $\omega_{I}$ was obtained from the data provided by Chulalongkorn Hospital [24]; $m_{i}$ was obtained from the UK Health Security Agency briefing [25]; β, v, and $d_{I}$ were obtained by model fitting. Therefore, the parameters used in this study were derived from the assumptions, calculations, and model adjustments as listed in Table 2. With the parameters in Table 2, the basic reproductive number in Thailand was calculated as $R_{0}$= 1.018691.

## 4. Effect of Vaccination on Disease Dynamics

**Figure 3:**
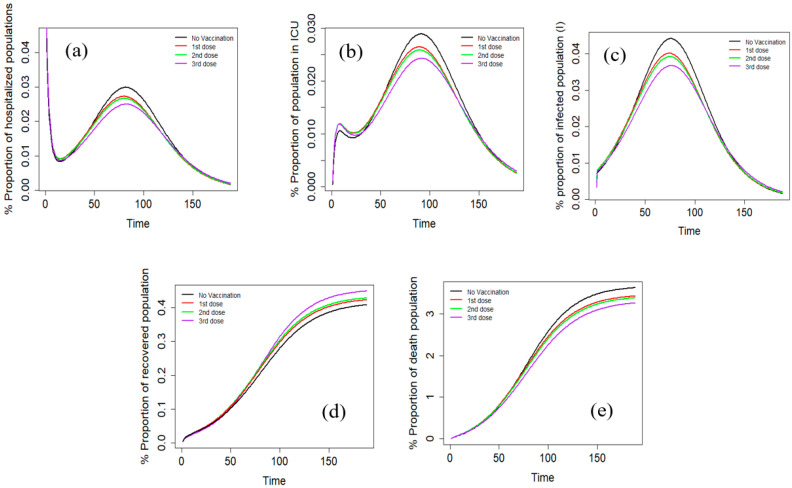
The effect of vaccination dose on hospitalized population (**a**), the infected population in ICU (**b**), the total infected population (**c**) recovered population (R) (**d**), and the percentage of the deaths (**e**).

The effects of different vaccination doses on the COVID-19 outcomes were also studied (Figure 3). It can be seen that vaccination significantly decreased the percentage of infected individuals, as well as hospitalization and mortality rates. These effects can be attributed to the ability of vaccines to stimulate the immune system and generate an effective response against the virus. Moreover, it is also clear that the impact of vaccination on infection rates varied depending on the number of doses received. The difference between the first and second doses was not significant, while there was a noticeable decline in the proportion of infected people after the third dose. Interestingly, the timing of the peak for infections and hospitalizations did not differ significantly between those who received one, two, or three vaccine doses. This suggests that the overall effect of vaccination is to reduce the severity of illness rather than alter the timing of the disease progression. It is important to note that the timing of the peak may depend on other factors, such as the level of virus transmission in the community and the effectiveness of non-pharmaceutical interventions.

In terms of survival and mortality, the study found that those who received vaccinations had better recovery rates than those who did not. Additionally, the death rate was lowest among those who received the third dose. This supports the notion that multiple doses of vaccination can provide additional protection against severe outcomes of the disease. Finally, the study found that the peak periods of ICU and hospital admission were longer for individuals who received one or two vaccine doses than those who received the third dose. This may be due to the increased protection provided by the third dose, which may lead to faster recovery and shorter hospital stays. Of note, in the case of COVID-19 vaccines, booster doses are intended to provide additional protection against the SARS-CoV-2 virus and its variants. The initial vaccine doses provide a strong immune response against the virus, but over time the protection provided by the vaccine may decline due to various factors, such as waning antibody levels or the emergence of new virus variants that are less susceptible to the immune response generated by the original vaccine. Booster doses are designed to re-stimulate the immune system’s memory of the initial response generated by the primary doses of the vaccine. By introducing an additional antigen, the booster dose can enhance and extend the body’s immune response against the virus. Overall, these findings provide valuable insights into the effectiveness of vaccination in reducing the severity of COVID-19 and suggest that multiple doses of vaccination may provide additional benefits in terms of reducing hospitalization and mortality rates.

## 5. Effective Reproduction Number and Simulation Outcome

**Figure 4:**
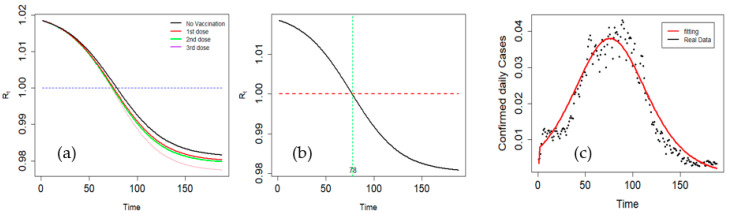
The effect of each vaccination dose on Rt (**a**), effective reproduction number (Rt) over time (**b**), and confirmed cases versus model fitting (**c**).

If the susceptible population varies over time, it is preferable to use the effective reproduction number (Rt). It is the average number of secondary cases arising from an infected case at a given point during the epidemic. Table 3 lists the vaccination rates and efficacies of the different doses. The vaccination rates were marginally higher at dose two but lower at dose three. Vaccination efficacy, however, improved dramatically after the third dose; of note, $m_{1}$,$m_{2}$ and $m_{3}$ were 0.58, 0.64, and 0.92, respectively. The effects of each dose on Rt are shown in Figure 4a. There was an apparent decrease in Rt with increasing dose. The results show that Rt decreases with time and starts to cross the threshold on day 78 after the start of wave 5. The findings of our simulation show that the pandemic will eventually fade under the current control, with a decline beginning on day 78, which corresponds to Rt < 1 (Figure 4b). Figure 4c shows the daily number of new active cases in Thailand and the numerical results of the proposed model. Between days 75 and 95, the daily increase in confirmed COVID-19 cases peaked. Our model predicts a peak during the same period. After 78 days, the number of cases steadily declined. These results confirm the suitability of the model for confirmed cases of COVID-19, as shown in Figure 4c.

## 6. Discussion

This study employed a mathematical model to assess the efficacy of vaccination during the fifth wave of the COVID-19 outbreak in Thailand. The model incorporated key dynamic factors of COVID-19, specifically the transmission originating from infected individuals and the mitigation arising from vaccination. The modeling results indicated that vaccination effectively reduced the spread of the virus during the fifth wave. Moreover, the findings underscored the importance of receiving multiple vaccine doses in reducing COVID-19 cases and fatalities. Specifically, individuals who received three vaccine doses exhibited a lower infection incidence than those who received only one or two doses. These results further reinforce the widely accepted notion that a two- or three-dose vaccine regimen provides enhanced protection against COVID-19.

More than 200 studies are available on compartment-based modeling of COVID-19; however, most of these studies lack the effect of vaccination [15]. As shown in Table 4, the booster dose was not included in most previous studies. In a previous study in Thailand, Riyapan et al. used a seven-compartment model and concluded that consistent use of face masks could play a significant role in managing the pandemic [26]. Although this model could generate peaks obtained from the actual data, there was no close overlap in the peak onset. Similarly, in a recent study in Thailand, Photphanlo et al. included both low- and high-risk groups and both pharmaceutical and non-pharmaceutical measures [27]. Although their model was very promising, comparison with real-world data was not evident, and the authors did not present vital parameters, such as specific analysis for hospitalization, recovery, and deaths, as in our study. Suphanchaimat et al. did include booster vaccine doses, but their study, though it made impressive forecasts, lacked the effect of individual vaccine doses and validation with real-world data [28]. In our study, keeping the model as simple and straightforward as possible was a primary concern because mathematical models are known to use a plethora of convoluted and superfluous parameters and equations. We examined the effects of vaccination separately for each of the five compartments (I, H, C, R, and D), regardless of whether there was no vaccination, one dose, two doses, or three doses. Despite comparable patterns at both ends, the peak region between the vaccinated and unvaccinated individuals revealed significant disparities. Similar trends are observed for I, H, C, and D. With both the presence and lack of vaccination, the number of affected people increased simultaneously. The model is theoretically examined to demonstrate the existence and positive invariance of the system solutions and to identify DFE and EE. We used the next-generation matrix method to obtain the whole reproduction number, $R_{0}$, and we examined the local stability of the DFE. We parameterized our model for Thailand using data from 3 January to 9 July 2022. We predicted daily confirmed cases using numerical simulations and data analyses and compared the model predictions with the existing data. Our model predicted that the percentage of vaccinated people who were unwell, severely ill, hospitalized, or died in the fifth wave would be lower, whereas the percentage of vaccinated people who had recovered would be higher. Here, we underline our theoretical findings from Theorem 1, which imply that the DFE is stable if Rt < 1. This result was in agreement with the simulation results. In our model, we highlight that vaccinated individuals may be infected with or without symptoms.

By the fifth phase of COVID-19, approximately 81% of the Thai population received at least one vaccination, 73% received two vaccinations, and 36% received a third vaccination [29]. However, coverage has been inconsistent throughout Thailand, with much lower vaccination rates among people living in remote areas [23]. The way people feel about vaccinations plays a direct role in the transmission of infections and their development into epidemics [24,30]. Moreover, there is a possibility that a section of the population did not receive vaccination because of skepticism regarding the efficacy and side effects of the vaccine [31]. Our findings, which show that three vaccine doses significantly minimized the number of negative outcomes related to COVID-19, are exciting and can help spread positive sentiments regarding booster doses. It is vital to remember that a significant proportion of people worldwide continue to resist vaccinations. In Thailand, in the fourth wave, 77% of patients received the first dose of the vaccine, 70% received the second dose, and 11% received the third dose. In the fifth wave, 81% of the participants received the first dose, 77% the second dose, and 42% the third dose. The Thai government must continue to increase vaccination rates to maintain the health of its tourism industry. High vaccination rates will greatly reduce the effective reproduction number. For instance, despite its high transmission rate, Saudi Arabia has an effective reproduction rate of less than 1, which may be attributed to its high immunization rate and booster dose [32]. In Thailand, public health strategies that include the ongoing monitoring of viral variants are also crucial. New variants may be immune to blood-based neutralizing antibodies, but vaccination is still effective in preventing serious diseases and death. It is unclear whether the Thai population has access to and is open to vaccinations for children. Furthermore, developing a reliable method to estimate how often booster shots are necessary will help plan and address the concerns of the general population regarding the financial and moral costs of the chosen government policy.

**Table 4:** Different studies on mathematical modeling of vaccination and COVID-19 progression.

Ref	Control	Measure Model	Country
[32]	Vaccination	SEIR + Quarantined + Hospitalized SAIR	Saudi Arabia
[33]	Full dose vaccination	SIR	Bangladesh
[34]	Vaccination	two-strain mode/SEIARD	USA
[35]	Vaccination	SEIR+ Asymptotic	Senegal
[36]	Double dose vaccination	SEIR	Ghana
[37]	Quarantine with treatment,	SEIR	Ethiopia
[38]	Double dose vaccination	SEIR	Tanzania
[27]	Low- and high-risk populations with pharmaceutical and non-pharmaceutical measures	SEIR+ vaccination	Thailand
This study	Double dose vaccination + Booster dose	SEIR+ vaccination	Thailand

As with any compartmental modeling framework, the present study has certain inherent limitations. For instance, the rate of vaccine ineffectiveness incorporated in the model is an average value for the entire population, which can differ depending on various demographic and clinical factors, as well as vaccine-related aspects such as age, type of vaccine administered, and the strain of the virus. Therefore, the accuracy of the findings could be improved by utilizing more granular data on the vaccine effectiveness rate for specific subgroups of the population. Additionally, the current understanding of the immunological response of humans to emerging SARS-CoV-2 variants and cross-immunity is limited. Consequently, there may be uncertainties in the model’s ability to accurately predict the effectiveness of the vaccine for new strains of the virus, particularly given the rapid emergence of new variants globally. It is also important to highlight that other approaches, such as linear algebra, can also solve this model. Exploring alternative approaches to yield the highest efficiency, versatility, robustness, and accuracy will be interesting. Future studies may provide a more comprehensive analysis of vaccine efficacy for specific subgroups, taking into account the evolving nature of the virus and emerging variants, which could improve the performance and accuracy of the model. For example, certain pre-existing medical conditions, age groups, and socioeconomic factors may contribute to higher rates of COVID-19 infection and mortality, but these factors may not be fully captured in compartmental models. An in-host viral infection model with time-inhomogeneous rates could be more effective in capturing different phases of the pandemic. Overall, while the modeling approach employed in this study was useful in elucidating the effectiveness of the vaccine during the fifth wave of the COVID-19 outbreak in Thailand, there is still scope for improvement in terms of accounting for the complexities associated with the immunological response of individuals and the evolution of the virus. Thus, further research is necessary to refine the model’s assumptions and enhance its ability to provide accurate predictions of vaccine effectiveness.

However, the major strength of our model lies in this degree of simplicity. The adaptable structure of the model makes the modeling of vaccines and immunological responses straightforward. We were able to predict the different effects of the different vaccine doses. There was almost a small processing overhead associated with running the model. Additionally, the versatility of our model lies in its ability to provide a wide range of solutions for critical parameters, including populations in hospitalized, recovered, and intensive care units. In future work, the model developed in this study will be used for COVID-19 outbreaks in other nations, and additional factors, such as viral strain, vaccination efficacy, age, and population diversity, will be included in the model.

## 7. Implications

Vaccination, particularly the booster dose, has been a subject of debate, and still, ambiguity persists regarding the beneficial effect of the booster dose. Moreover, although several mathematical models have been proposed for COVID-19 progression, their applicability to real-world data has been an area of concern, mainly because of the emergence of new variants and heterogeneous prevention measures such as masks, vaccinations, and social distancing, which are difficult to estimate reliably and thus lead to variability in the model outcome.

This study presented a simple and efficient model with the potential to accurately predict disease progression and several outcomes of interest. Our model was a good fit for the COVID-19 fifth-wave data from Thailand and established the effectiveness of the vaccination and booster doses. These results will help clinicians, policymakers, and the general public to have a more informed assessment of COVID-19 progression dynamics and vaccination effectiveness.

Further research is required to test the generalizability of our model under diverse circumstances. Particular attention should be paid to factoring in the virulence of novel variations and vaccination efficacies as well as capturing such dynamic aspects in mathematical models.

## 8. Conclusions

We introduced a new seven-compartment mathematical model for COVID-19 that considers the dynamics of propagation in vaccinated and unvaccinated populations. Our results confirmed the beneficial effects of vaccines in terms of infection control and reduction of the adverse effects of COVID-19. The results showed that vaccination is vital to reduce COVID-19 and the pandemic in Thailand. A similar trend was observed in the proportion of hospitalized patients and those in the intensive care unit (ICU). In particular, the dose administered did not significantly alter the peak time. There was an apparent decrease in Rt with an increase in dose, and Rt crossed the threshold on day 78 after the start of the fifth wave. We provided an analytical expression for the effective reproduction number, which is a critical factor in determining the necessary conditions for endemic and DFE. Our theoretical findings and numerical analysis were applied to the fifth wave of the pandemic in Thailand, and a good overlap between real-world and simulated data was obtained. We offered mathematical proof that the vaccine reduced the transmission rate, and our results indicate that the vaccine helps reduce infections, hospitalizations, and critical conditions. We recommend that more studies be conducted to capture the uncertainty inherent in the dynamic nature of the disease and incorporate risk assessment.

## Figures and Tables

**Table 1:** Summary of model parameters and descriptions.

Parameters	
$m_{i}$	Vaccine efficiency of ith dose of vaccination
β	Infection rate
$\tau_{i}$	Vaccination rates of ith dose of vaccination
v	Vaccine ineffectiveness rate
$\gamma_{I}$	Recovery from infection
$\gamma_{H}$	Recovery from infection while in hospital
$\omega_{I}$	Hospital admission rate
$\omega_{H}$	ICU admission rate of infected hospitalized individuals
$\gamma_{C}$	The recovery rate from infection in the ICU, returning to the hospital
$d_{C}$	The death rate of the population admitted to the ICU.
$d_{I}$	The death rate of the infected population
$d_{H}$	The death rate of the hospitalized population

**Table 2:** Values of model parameters corresponding to COVID-19 cases in Thailand.

Parameters		Value	Unit	Source
$m_{i}$	Vaccine efficacy		%	[25]
β	Infection rates in the population	1.95	Per day	fitted
$\tau_{i}$	Vaccination rates of risk populations		Per day	[22]
v	Vaccine ineffectiveness rate	8.55	Per day	fitted
$\gamma_{I}$	Rate of recovery from infection		Per day	[22,23]
$\gamma_{H}$	Rate of recovery from infection	1/10	Per day	[22,23]
$\omega_{I}$	Rates of hospital admissions from infected groups	1/3	Per day	[24]
$\omega_{H}$	The rate of admission to the ICU from the group of infected people admitted to the hospital	1/10	Per day	[4,23]
$\gamma_{C}$	The rate of recovery from infection in the ICU, returning to the hospital	1/20	Per day	[22]
$d_{C}$	Death rate of the population admitted to the ICU.	1/20	Per day	[22,23]
$d_{I}$	Death rate of the infected population	1.51	Per day	fitted
$d_{H}$	Death rate of the hospitalized population	1/10	Per day	[22,23]

**Table 3:** Vaccination rates and efficacy at different doses.

Parameters	Value	Parameters	Value
$\tau_{0}$	0	$m_{0}$	0
$\tau_{1}$	0.004574	$m_{1}$	0.58
$\tau_{2}$	0.004966	$m_{2}$	0.64
$\tau_{3}$	0.00299	$m_{3}$	0.92

## Data Availability

Not applicable.

## Abbreviations

DDC	Department of Disease Control
DFE	Disease-free equilibrium
EEP	Endemic equilibrium point
ICU	Intensive care unit
SIR	Susceptible-infected-removal
TSRI	Thailand Science Research and Innovation

## Appendix A

This appendix provides technical details supporting the analysis in the manuscript.

### Appendix A.1. Proof of Theorem 1

By equating all derivatives mentioned in the set of the system in model (1) to zero and then solving the equations considering all components being positive, we get

(A1) $$0=vS_{v}-\beta SI-\tau_{i}S$$

(A2) $$0=\tau_{i}S-(1-m_{i})\beta S_{v}I-vS_{v}$$

(A3) $$0=\beta SI+(1-m_{i})\beta S_{v}I-\gamma_{I}I-\omega_{I}I-d_{I}I$$

(A4) $$0=\omega_{I}I+\gamma_{C}C-\omega_{H}H-\gamma_{H}H-d_{H}H$$

(A5) $$\omega_{H}H-\gamma_{C}C-d_{C}C$$

(A6) $$0=\gamma_{I}I+\gamma_{H}H$$

(A7) $$0=d_{I}I+d_{H}H+d_{C}C$$

and

(A8) $$S_{v}+S+I+H+C+R+D=1$$

At disease-free equilibrium, we have $\left(S^{*},S_{v}^{*},I^{*},H^{*},C^{*},R^{*},D^{*}\right)=\left(S^{*},S_{v}^{*},0,0,0,0,0\right)$

(A9) $$0=vS_{v}-\beta SI-\tau_{i}S$$

(A10) $$0=\tau_{i}S-(1-m_{i})\beta S_{v}I-vS_{v}$$

From (A8) and $I^{*},H^{*},C^{*},R^{*},D^{*}=0$, we have

$$S_{v}+S=1$$

Then

$$S=1-S_{v}$$

Substitute $S=1-S_{v}$ in (9), and we get

$$vS_{v}-\beta \left(1-S_{v}\right)I-\tau_{i}\left(1-S_{v}\right)=0$$

Rearranging above equation, we have

$$S_{v}=\frac{\tau_{i}(1-S_{v})}{v}=\frac{\tau_{i}S}{v}$$

$$S=\frac{{vS}_{v}}{\tau_{i}}$$

Therefore, $\left(S^{0},S_{v}^{0},I^{0},H^{0},C^{0},R^{0},D^{0}\right)=\left(S^{0}=\frac{{vS}_{v}^{0}}{\tau_{i}},S_{v}^{0}=S_{v}^{0},0,0,0,0,0\right)$.

Similarly, for the endemic equilibrium, we equate all derivatives mentioned in the set of the system in model (1) to zero which become Equations (A1)–(A7) and then solve the equations considering all components being positive.

From (A8), we get

(A11) $$D=1-(S_{v}+S+I+H+C+R)$$

Take (A4) + (A6), we get

$$0=\omega_{I}I+\gamma_{C}C-\omega_{H}H-d_{H}H+\gamma_{I}I=\left(\omega_{I}+\gamma_{I}\right)I+\gamma_{c}C-\left(\omega_{H}+d_{H}\right)H$$

Thus, $H^{*}=\frac{\left(\omega_{I}+\gamma_{I}\right)I^{*}+\gamma_{c}C^{*}}{\left(\omega_{H}+d_{H}\right)}$

Therefore, we have endemic equilibrium as $\left(S^{*},S_{v}^{*},I^{*},H^{*},C^{*},R^{*})=(S^{*},S_{v}^{*},I^{*},H^{*}=\frac{(\omega_{I}+\gamma_{I})I^{*}+\gamma_{c}C^{*}}{\left(\omega_{H}+d_{H}\right)},C^{*},R^{*}\right)$ where $S^{*},S_{v}^{*},I^{*},C^{*}$,$R^{*}$ > 0 are arbitrary.

### Appendix A.2. Proof of Theorem 3

The Jacobian matrix of model (1) evaluated at $E^{*}$ is

$$J_{E^{*}}=\left[\begin{array}{ccccc} -(\beta +\tau_{i}) & v & -\beta S^{*} & 0 & 0\\ \tau_{i} & -(1-m_{i})\beta I^{*}-v & -(1-m_{i})\beta S_{v}^{*} & 0 & 0\\ \beta I^{*} & (1-m_{i})\beta I^{*} & \beta S^{*}+(1-m_{i})\beta S_{v}^{*}-(\gamma_{I}+\omega_{I}+d_{I}) & 0 & 0\\ \begin{array}{c} 0\\ 0 \end{array} & \begin{array}{c} 0\\ 0 \end{array} & \begin{array}{c} \omega_{I}\\ 0 \end{array} & \begin{array}{cc} \begin{array}{c} -(\omega_{H}+\gamma_{H}+d_{H})\\ \omega_{H} \end{array} & \begin{array}{c} \gamma_{C}\\ -(\gamma_{C}+d_{C}) \end{array} \end{array} \end{array}\right]$$

$$\det(J_{E^{*}}-\lambda I)=\left[\begin{array}{ccccc} -(\beta I^{*}+\tau_{i})-\lambda_{1} & v & -\beta S^{*} & 0 & 0\\ \tau_{i} & -(\left(1-m_{i}\right)\beta +v)-\lambda_{2} & -(1-m_{i})\beta S_{v}^{*} & 0 & 0\\ \beta I^{*} & (1-m_{i})\beta I^{*} & \beta S^{*}+(1-m_{i})\beta S_{v}^{*}-(\gamma_{I}+\omega_{I}+d_{I})-\lambda_{3} & 0 & 0\\ \begin{array}{c} 0\\ 0 \end{array} & \begin{array}{c} 0\\ 0 \end{array} & \begin{array}{c} \omega_{I}\\ 0 \end{array} & \begin{array}{cc} \begin{array}{c} -(\omega_{H}+\gamma_{H}+d_{H})-\lambda_{4}\\ \omega_{H} \end{array} & \begin{array}{c} \gamma_{C}\\ -(\gamma_{C}+d_{C})-\lambda_{5} \end{array} \end{array} \end{array}\right]$$

We get

$$\begin{array}{ll} (\lambda^{2}+(d_{c}+d_{H}+ & \gamma_{C}+\gamma_{H}+\omega_{H})\lambda +d_{c}d_{H}+d_{c}\gamma_{H}+d_{c}\omega_{H}+d_{H}\gamma_{C}+\gamma_{C}\gamma_{H})(-\lambda^{3}\\ & -\lambda^{2}((d_{I}+\gamma_{I}+\omega_{I}+\tau_{i}+v)-\beta (\left(1-m)(S_{v}-I)+(S-I\right)))\\ -\lambda \{\left(1-m_{i}\right)\beta (-I^{2}\beta +I\beta S+ & IS_{v}\beta -I\tau_{i}+S_{v}\tau_{i}+S_{v}v-I{(d}_{I}+\gamma_{I}+\omega_{I})-I\beta {(d}_{I}+\gamma_{I}+\omega_{I})-I\beta v+S\beta \tau_{i}+\\ & S\beta v-\tau_{i}(d_{I}+\gamma_{I}+\omega_{I})-{v(d}_{I}+\gamma_{I}+\omega_{I})\}\\ +{(1-m)(-I}^{2}\beta^{2}d_{I}-I^{2} & \beta^{2}\gamma_{I}-I\beta d_{I}\tau_{i}-I\beta \gamma \tau_{i}-I\beta \omega_{I}\tau_{i}-I^{2}\beta^{2}\omega_{I})-I\beta {v(d}_{I}+\gamma_{I}+\omega_{I}) \end{array}$$

The equation can be expressed as $\left(\lambda^{2}+b\lambda +c\right)(-1)\left(\lambda^{3}+e\lambda^{2}+f\lambda +(-g\right))=0$, where

$$b=d_{c}+d_{H}+\gamma_{C}+\gamma_{H}+\omega_{H}>0$$

$$c=d_{c}d_{H}+d_{c}\gamma_{H}+d_{c}\omega_{H}+d_{H}\gamma_{C}+\gamma_{C}\gamma_{H}>0$$

According to the Routh–Hurwitz criteria [39], the first term has negative real parts.

Consider the second term that can be expressed as $\left(\lambda^{3}+e\lambda^{2}+f\lambda +(-g\right))$

We have

$$e=\left(d_{I}+\gamma_{I}+\omega_{I}+\tau_{i}+v\right)-\beta \left(\left(1-m_{i}\right)\left(S_{v}-I\right)+\left(S-I\right)\right)$$

$$f=\left(1-m_{i}\right)\beta (-I^{2}\beta +I\beta S+IS_{v}\beta -I\tau_{i}+S_{v}\tau_{i}+S_{v}v-I{(d}_{I}+\gamma_{I}+\omega_{I}))-I\beta {(d}_{I}+\gamma_{I}+\omega_{I})-I\beta v+S\beta \tau_{i}+\;S\beta v-\tau (d_{I}+\gamma_{I}+\omega_{I})-{v(d}_{I}+\gamma_{I}+\omega_{I})$$

$$-g=-(\left(1-m_{i}\right)I\beta (-I\beta d_{I}-I\beta \gamma_{I}-d_{I}\tau_{i}-\gamma \tau_{i}-\omega_{I}\tau_{i}-I\beta \omega_{I})-I\beta {v(d}_{I}+\gamma_{I}+\omega_{I}))$$

According to the Routh–Hurwitz criteria [39], we need to show that e>0,g>0 and ef>g so that the solution of the polynomial has negative real parts.

Consider $e=\left(d_{I}+\gamma_{I}+\omega_{I}+\tau_{i}+v\right)-\beta \left(\left(1-m_{i}\right)\left(S_{v}-I\right)+\left(S-I\right)\right)$

Given the condition that $S<I-\left(1-m_{i}\right){(S}_{v}-I)andI>S_{v}$, we have

$$e=\left(d_{I}+\gamma_{I}+\omega_{I}+\tau_{i}+v\right)-\beta \left(\left(1-m\right)\left(S_{v}-I\right)+\left(S-I\right)\right)>0$$

Now consider

$$-g=-(\left(1-m_{i}\right)I\beta (-I\beta d_{I}-I\beta \gamma_{I}-d_{I}\tau_{i}-\gamma \tau_{i}-\omega_{I}\tau_{i}-I\beta \omega_{I})-I\beta {v(d}_{I}+\gamma_{I}+\omega_{I}))\;=\left(1-m_{i}\right)I\beta \left(I\beta \left(d_{I}+\gamma_{I}+\omega_{I}\right)+\tau_{I}\left(d_{I}+\gamma_{I}+\omega_{I}\right)\right)+I\beta {v(d}_{I}+\gamma_{I}+\omega_{I})$$

Hence, −g>0,

We want to show that ef>−g, which means we need to prove that ef+g>0

We used Maple 2018 to help us calculate the algebraic expressions, and for convenience we set $W=d_{I}+\gamma_{I}+\omega_{I}$ and $N=\tau_{i}+v$, so we have

$$ef=((1-m_{i})\beta (-I^{2}\beta +IS\beta +IS_{v}\beta -IW-I\tau_{i}+NS_{v})-WI\beta -WN-I\beta v\;+S\beta N)(W+N-\beta ((1-m_{i})(S_{v}-I)+S-I))$$

$$\begin{array}{ll} ef+g=\beta (-I^{2}\beta & +IS\beta +IS_{v}\beta -IW-I\tau_{i}+NS_{v})N+\beta (-I^{2}\beta +IS\beta +IS_{v}\beta -IW-I\tau_{i}\\ & +NS_{v})W-WN^{2}-W^{2}N+WI\beta^{2}S+WI\beta^{2}S_{v}-3WI\beta N+2WNS\beta +WNS_{v}\beta \\ & +I^{2}\beta^{2}vm_{i}+I\beta^{2}vS-2I\beta vW+I\beta^{2}vS_{v}-I\beta vN+2S\beta^{2}NI-S\beta^{2}NS_{v}\\ & -IW\beta \tau_{i}-3\beta \left(-I^{2}\beta +IS\beta +IS_{v}\beta -IW-I\tau_{i}+NS_{v}\right)I\beta m_{i}\\ & +2\beta \left(-I^{2}\beta +IS\beta +IS_{v}\beta -IW-I\tau_{i}+NS_{v}\right)S_{v}\beta m_{i}\\ & +\beta \left(-I^{2}\beta +IS\beta +IS_{v}\beta -IW-I\tau_{i}+NS_{v}\right)m_{i}^{2}I\beta \\ & -\beta \left(-I^{2}\beta +IS\beta +IS_{v}\beta -IW-I\tau_{i}+NS_{v}\right)m_{i}^{2}S_{v}\beta \\ & +\beta \left(-I^{2}\beta +IS\beta +IS_{v}\beta -IW-I\tau_{i}+NS_{v}\right)m_{i}S\beta \\ & +2\beta \left(-I^{2}\beta +IS\beta +IS_{v}\beta -IW-I\tau_{i}+NS_{v}\right)I\beta \\ & -\beta \left(-I^{2}\beta +IS\beta +IS_{v}\beta -IW-I\tau_{i}+NS_{v}\right)S_{v}\beta -W^{2}I\beta +2\beta^{2}Wm_{i}I^{2}\\ & -WI\beta^{2}S_{v}m_{i}+WNI\beta m_{i}-WNS_{v}\beta m_{i}-I\beta^{2}vS_{v}m_{i}-S\beta^{2}NIm_{i}+S\beta^{2}NS_{v}m_{i}\\ & +IW\beta m_{i}\tau_{i}-\beta \left(-I^{2}\beta +IS\beta +IS_{v}\beta -IW-I\tau_{i}+NS_{v}\right)S\beta \\ & -\beta \left(-I^{2}\beta +IS\beta +IS_{v}\beta -IW-I\tau_{i}+NS_{v}\right)m_{i}N-S^{2}\beta^{2}N+S\beta N^{2}-2I^{2}\beta^{2}v\\ & -\beta \left(-I^{2}\beta +IS\beta +IS_{v}\beta -IW-I\tau_{i}+NS_{v}\right)m_{i}W-3\beta^{2}WI^{2} \end{array}$$

For convenience, we let $F=\beta \left(-I^{2}\beta +IS\beta +IS_{v}\beta -IW-I\tau_{i}+NS_{v}\right)$

$$\begin{array}{ll} ef+g=FN+FW & -WN^{2}-W^{2}N+WI\beta^{2}S+WI\beta^{2}S_{v}-3WI\beta N+2WNS\beta +WNS_{v}\beta +I^{2}\beta^{2}vm_{i}+I\beta^{2}vS\\ & -2I\beta vW+I\beta^{2}vS_{v}-I\beta vN+2S\beta^{2}NI-S\beta^{2}NS_{v}-IW\beta \tau_{i}-3FI\beta m_{i}+2FS_{v}\beta m_{i}+Fm_{i}^{2}I\beta \\ & -Fm_{i}^{2}S_{v}\beta +Fm_{i}S\beta +2FI\beta -FS_{v}\beta -W^{2}I\beta +2\beta^{2}Wm_{i}I^{2}-WI\beta^{2}S_{v}m_{i}+WNI\beta m_{i}\\ & -WNS_{v}\beta m_{i}-I\beta^{2}vS_{v}m_{i}-S\beta^{2}NIm_{i}+S\beta^{2}NS_{v}m_{i}+IW\beta m_{i}\tau_{i}-FS\beta -Fm_{i}N-S^{2}\beta^{2}N\\ & +S\beta N^{2}-2I^{2}\beta^{2}v-Fm_{i}W-3\beta^{2}WI^{2} \end{array}$$

$$\begin{array}{ll} ef+g=\left(1-m_{i}\right) & FN+\left(1-m_{i}\right)FW-WN^{2}-W^{2}N+WI\beta^{2}S+\left(1-m_{i}\right)WI\beta^{2}S_{v}+\left(m_{i}-1\right)WNI\beta +(S\\ & -I)2WNS\beta +\left(1-m_{i}\right)WNS_{v}\beta +I\beta^{2}vS-2I\beta vW+\left(1-m_{i}\right)I\beta^{2}vS_{v}-I\beta vN\\ & +\left(1-m_{i}\right)S\beta^{2}NI+IS\beta^{2}NI+(m_{i}-1)S\beta^{2}NS_{v}+(m_{i}-1)IW\beta \tau_{i}+\left(2-m_{i}\right)\left(1-m_{i}\right)FI\beta \\ & +(2-m_{i})(1+m_{i})FS_{v}\beta -FS_{v}\beta -W^{2}I\beta +(m_{i}-1)Fm_{i}S\beta +(N-S\beta )S\beta N\\ & +{(m_{i}-2)I}^{2}\beta^{2}v+(2m_{i}-3)\beta^{2}WI^{2} \end{array}$$

If $m_{i}=1$ and $\tau_{i}=0,v=0$, then we have

(A12) $$\begin{array}{ll} ef+g & =WI\beta^{2}S+2FS_{v}\beta -FS_{v}\beta -W^{2}I\beta -\beta^{2}WI^{2}\\ & =WI\beta^{2}S+FS_{v}\beta -W^{2}I\beta -\beta^{2}WI^{2}\\ & =WI\beta^{2}S-\beta^{3}I^{2}+IS\beta^{3}+IS_{v}\beta^{3}-IW\beta^{2}-W^{2}I\beta -\beta^{2}WI^{2} \end{array}$$

If F>0, then $\beta \left(-I^{2}\beta +IS\beta +IS_{v}\beta -IW-I\tau_{i}+NS_{v}\right)$ = ${-\beta }^{2}I^{2}+IS\beta^{2}+IS_{v}\beta^{2}-IW\beta -I\tau_{i}\beta +NS_{v}\beta >0$

Note that $\tau_{i}=0,v=0$, we then get

(A13) $$IS\beta^{2}+IS_{v}\beta^{2}>\beta^{2}I^{2}+IW\beta$$

Hence, from (A13), we multiply β with both sides, and we get

(A14) $$\left(IS\beta^{3}+IS_{v}\beta^{3}\right)>\beta^{3}I^{2}+IW\beta^{2}$$

From (A12), we get

$$\begin{array}{ll} ef+g= & WI\beta^{2}S-\beta^{3}I^{2}+(IS\beta^{3}+IS_{v}\beta^{3})-IW\beta^{2}-W^{2}I\beta -\beta^{2}WI^{2}\\ & >WI\beta^{2}S-\beta^{3}I^{2}+\left(\beta^{3}I^{2}+IW\beta^{2}\right)-IW\beta^{2}-W^{2}I\beta \\ & -\beta^{2}WI^{2}=WI\beta^{2}S-W^{2}I\beta -\beta^{2}WI^{2}=WI\beta \left(\beta S-\beta I-W\right) \end{array}$$

Given that other parameter values are not equal to zero, term WIβ(βS−βI−W) = 0 if and only (βS−βI−W) = 0

As a result,

(A15) $$S>\frac{\beta I+(d_{I}+\gamma_{I}+\omega_{I})}{\beta }$$

Consider again F>0, and we have

$$S\beta +S_{v}\beta >\beta I+\left(d_{I}+\gamma_{I}+\omega_{I}\right)=S\beta$$

$S_{v}\beta >0$, which means $S_{v}>0.$

Thus, if $R_{0}>1,$ then the endemic equilibrium point, $E^{*}$ of the model (1) is stable under the condition that $0<S_{v}<I$ where $\frac{\beta I+(d_{I}+\gamma_{I}+\omega_{I})}{\beta }<S<I-(1-m_{i}){(S}_{v}-I).$

## References

[1] BostonGlobe A New Coronavirus Variant Has Taken Over, Sparking Concerns of A Winter Surge—The Boston Globe. https://www.bostonglobe.com/2022/11/21/nation/new-coronavirus-variant-has-taken-over-sparking-concerns-winter-surge/.

[2] Sarun C., Craven M., Lamb J., Sabow A., Singhal S., Wilson M. When Will the COVID-19 Pandemic End?. https://www.mckinsey.com/industries/healthcare-systems-and-services/our-insights/when-will-the-covid-19-pandemic-end.

[3] WHO One Year Since The Emergence of COVID-19 Virus Variant Omicron. https://www.who.int/news-room/feature-stories/detail/one-year-since-the-emergence-of-omicron.

[4] Google News Coronavirus (COVID-19)—Google News. https://news.google.com/covid19/map?hl=en-IN&gl=IN&ceid=IN%3Aen.

[5] Kyriakidis N.C., Lopez-Cortes A., Gonzalez E.V., Grimaldos A.B., Prado E.O. (2021). SARS-CoV-2 vaccines strategies: A comprehensive review of phase 3 candidates. NPJ Vaccines.

[6] Cascella M., Rajnik M., Aleem A., Dulebohn S.C., Di Napoli R. (2022). Features, Evaluation, and Treatment of Coronavirus (COVID-19).

[7] Ritchie H., Mathieu E., Rodés-Guirao L., Appel C., Giattino C., Ortiz-Ospina E., Hasell J., Macdonald B., Beltekian D., Roser M. Coronavirus Pandemic (COVID-19). https://ourworldindata.org/coronavirus..

[8] El-Elimat T., AbuAlSamen M.M., Almomani B.A., Al-Sawalha N.A., Alali F.Q. (2021). Acceptance and attitudes toward COVID-19 vaccines: A cross-sectional study from Jordan. PLoS ONE.

[9] Durmaz N., Suman M., Ersoy M., Orun E. (2022). Parents’ attitudes toward childhood vaccines and COVID-19 vaccines in a Turkish pediatric outpatient population. Vaccines.

[10] Almeshari M., Abanomy A., Alzamil Y., Alyahyawi A., Al-Thomali A.W., Alshihri A.A., Althomali O.W. (2022). Public acceptance of COVID-19 vaccination among residents of Saudi Arabia: A cross-sectional online study. BMJ Open.

[11] Kartono A., Karimah S.V., Wahyudi S.T., Setiawan A.A., Sofian I. (2021). Forecasting the Long-Term Trends of Coronavirus Disease 2019 (COVID-19) Epidemic Using the Susceptible-Infectious-Recovered (SIR) Model. Infect. Dis. Rep..

[12] Tolles J., Luong T. (2020). Modeling epidemics with compartmental models. JAMA.

[13] Abou-Ismail A. (2020). Compartmental models of the COVID-19 pandemic for physicians and physician-scientists. SN Compr. Clin. Med..

[14] Hoertel N., Blachier M., Blanco C., Olfson M., Massetti M., Rico M.S., Limosin F., Leleu H. (2020). A stochastic agent-based model of the SARS-CoV-2 epidemic in France. Nat. Med..

[15] Kong L., Duan M., Shi J., Hong J., Chang Z., Zhang Z. (2022). Compartmental structures used in modeling COVID-19: A scoping review. Infect. Dis. Poverty.

[16] Tantrakarnapa K., Bhopdhornangkul B., Nakhaapakorn K. (2022). Influencing factors of COVID-19 spreading: A case study of Thailand. J. Public Health.

[17] Pongsakornrungsilp S., Pongsakornrungsilp P., Kumar V., Maswongssa B. (2021). The art of survival: Tourism businesses in Thailand Recovering from COVID-19 through brand management. Sustainability.

[18] Shadmi E., Chen Y., Dourado I., Faran-Perach I., Furler J., Hangoma P., Hanvoravongchai P., Obando C., Petrosyan V., Rao K.D. (2020). Health equity and COVID-19: Global perspectives. Int. J. Equity Health.

[19] Diekmann O., Heesterbeek J.A., Roberts M.G. (2010). The construction of next-generation matrices for compartmental epidemic models. J. R. Soc. Interface.

[20] Wang W., Zhao X.-Q. (2008). Threshold dynamics for compartmental epidemic models in periodic environments. J. Dyn. Differ. Equ..

[21] van den Driessche P., Watmough J. (2002). Reproduction numbers and sub-threshold endemic equilibria for compartmental models of disease transmission. Math. Biosci..

[22] DDC DDC COVID-19 Interactive Dashboard 1-Dash-Tiles-W. https://ddc.moph.go.th/covid19-dashboard.

[23] Bangkokbiznews How Is the Situation of “COVID-19” Around the World after Facing “Omicron”?. https://www.bangkokbiznews.com/social/1000909.

[24] Chulalongkorn COVID-19 “Omicron”. https://chulalongkornhospital.go.th/kcmh/.

[25] UKHSA SARS-CoV-2 Variants of Concern and Variants under Investigation in England. https://eprints.whiterose.ac.uk/185514/.

[26] Riyapan P., Shuaib S.E., Intarasit A. (2021). A Mathematical Model of COVID-19 Pandemic: A Case Study of Bangkok, Thailand. Comput. Math. Methods Med..

[27] Ritraksa S., Photphanloet C., Shuaib S.E., Intarasit A., Riyapan P. (2023). Mathematical modeling to study the interactions of two risk populations in COVID-19 spread in Thailand. AIMS Math..

[28] Suphanchaimat R., Teekasap P., Nittayasoot N., Phaiyarom M., Cetthakrikul N. (2022). Forecasted trends of the new COVID-19 epidemic due to the Omicron variant in Thailand, 2022. Vaccines.

[29] Algarni A.D., Ben Hamed A., Hamdi M., Elmannai H., Meshoul S. (2022). Mathematical COVID-19 model with vaccination: A case study in Saudi Arabia. PeerJ Comput. Sci..

[30] Paul A.K., Kuddus M.A. (2022). Mathematical analysis of a COVID-19 model with double dose vaccination in Bangladesh. Results Phys..

[31] de Leon U.A., Avila-Vales E., Huang K.L. (2022). Modeling COVID-19 dynamic using a two-strain model with vaccination. Chaos Solitons Fractals.

[32] Diagne M.L., Rwezaura H., Tchoumi S.Y., Tchuenche J.M. (2021). A Mathematical Model of COVID-19 with Vaccination and Treatment. Comput Math. Methods Med..

[33] Akuka P.N.A., Seidu B., Bornaa C.S. (2022). Mathematical analysis of COVID-19 transmission dynamics model in Ghana with double-dose vaccination and quarantine. Comput. Math. Methods Med..

[34] Teklu S.W. (2022). Mathematical analysis of the transmission dynamics of COVID-19 infection in the presence of intervention strategies. J. Biol. Dyn..

[35] Ayoola T.A., Kolawole M.K., Popoola A.O. (2022). Mathematical model of COVID-19 transmission dynamics with double dose vaccination. Tanzan. J. Sci..

[36] Hfocus Thai COVID Is Not Yet Endemic. https://www.hfocus.org/content/2022/04/24967.

[37] KRUNGTHAI Comparison of COVID-19 Vaccines, Effectiveness and Side Effects. https://www.krungthai-axa.co.th/th/health-tip-compared-site-effects-of-the-covid-vaccine.

[38] NSO National Statistical Office Thailand. https://www.nso.go.th/sites/2014en.

[39] Gantmakher F.R. (2000). Matrix Theory.

